# Ageism on the Therapeutic Couch: Aging Attitudes and Late-Life Psychotherapy

**DOI:** 10.1093/geroni/igab046.1669

**Published:** 2021-12-17

**Authors:** Gregory Hinrichsen

**Affiliations:** Icahn School of Medicine at Mount Sinai, New York, New York, United States

## Abstract

Ageist stereotypes characterize older adults as depressed, demented, and dependent. A large body of research has documented the adverse physical and emotional impact of ageism on older adults. Mental health professionals, however, often see the minority of older adults who, in fact, are depressed, have cognitive impairments, and/or are increasingly dependent on others. To what degree do pre-existing attitudes about aging come into play in psychotherapy with older people? With all age groups, psychotherapists often help clients better understand and challenge longstanding negative assumptions about self and world (sometimes called “the unconscious” or “underlying schemas”). These assumptions often impede the individual’s ability to successfully contend with life problems. This presentation will discuss ways in which psychotherapists can assist older clients in clarifying their underlying (and often self-limiting) negative assumptions about aging, moving beyond them to better contend with late life stressors, and improving emotional well-being.

